# The effects of mixed-mode ventilation on energy saving and employee job satisfaction, work engagement, and job performance

**DOI:** 10.1038/s41598-026-38812-0

**Published:** 2026-02-12

**Authors:** Yamon Min Ye, Wei Liang, Fei Xu, Adrian Chong, Christopher M. Barnes, Kai Chi Yam

**Affiliations:** 1 Department of Management and Organisation, National University of Singapore, Singapore, Singapore; 2 Department of the Built Environment, National University of Singapore, Singapore, Singapore; 3 Department of Management and Organization, University of Washington, Seattle, USA

**Keywords:** Mixed-mode ventilation, Green technology, Job performance, Job satisfaction, Work engagement, Engineering, Environmental sciences, Environmental social sciences

## Abstract

Given the rise in extreme temperatures worldwide due to climate change, this paper explores whether installing a mixed-mode ventilation (MMV) system in buildings can reduce energy consumption without compromising employees’ job satisfaction, work engagement, and job performance. The MMV system combines natural ventilation with air-conditioning, switching between them as needed and operating at a higher temperature to achieve energy savings while still providing thermal comfort. A 19-week experience sampling study (Study 1) was conducted to examine the long-term effects of working under the MMV system. A field experiment (Study 2) was then conducted to document causal effects. The results show that the MMV system leads to significant energy savings compared to traditional air-conditioners (AC) and, furthermore, provide initial evidence that employees’ job satisfaction, work engagement, and job performance do not differ statistically between those working under the MMV system and those working under traditional AC.

**Supplementary Information:**

The online version contains supplementary material available at 10.1038/s41598-026-38812-0.

Developed economies have invested significant resources into studying the environmental impacts of major polluting factors such as vehicle emissions^1^, agriculture^2^, and diet^3^. Air conditioners (ACs) have thus far evaded scholarly attention, in part because most developed economies with four seasons only require ACs during summer. However, the Global South experiences higher temperatures throughout the year on average than their Global North counterparts. Moreover, the Global South is home to over 85% of the world’s population. As a result, most buildings in these countries are either air-conditioned or candidates for air conditioning. Unfortunately, ACs further contribute to climate change by directly emitting hot air outside and indirectly through enormous energy expenditure and greenhouse gas emissions^4,5^. This paper takes an interdisciplinary approach to tackle this issue, where two studies were conducted to examine the environmental, psychological, and behavioral impacts of a mixed-mode ventilation (MMV) system on employees.

This research joins an emerging interdisciplinary conversation among environmental psychologists and organizational psychologists on biophilic work design^6^. Specifically, past research has theorized and found that architecture and building design can enhance individual well-being and organizational outcomes^7–9^. A crucial factor predicting these important work outcomes is thermal comfort, defined as “the mental state of satisfaction with the thermal environment”^10^. Past research indicates that a comfortable thermal environment lowers fatigue^11^, induces positive emotions^11^, and ultimately leads to better work performance^11^. One study finds that reducing the temperature from 25.1 to 22.6 °C increased productivity by up to 8% and this increased productivity was sustained across 3 months^12^, while another study finds that percentage of direct work time decreases by 0.57% for every 1 °C increase in environmental temperature^13^, highlighting the importance of a comfortable thermal environment to worker productivity.

To provide thermal comfort to occupants, commercial buildings – where most organizations are located – typically rely on central ACs, leading to high energy consumption and carbon emissions. Although thermal comfort is crucial for employee outcomes, ACs further contribute to climate change by directly emitting hot air outside and indirectly through enormous energy expenditure and greenhouse gas emissions^4,5^. Fortunately, in the separate domain of building design and engineering, researchers have developed a potential solution that can address this issue by designing a mixed-mode ventilation (MMV) system that can provide thermal comfort while also consuming less energy. A traditional MMV system works by combining natural ventilation from operable windows with mechanical air-conditioning to provide thermal comfort to occupants while reducing energy consumption. It has been proven to reduce energy consumption while still providing thermal comfort^14^. However, due to its high reliance on natural ventilation, its use has been more limited in the tropical climates, where weather conditions tend to be hotter and more humid. Recent advances in MMV technology have allowed for the development of a MMV system that adopts multi-stage increment cooling approaches (details in Appendix A, Figs. 1, 2, and S1).

**Fig. 1:**
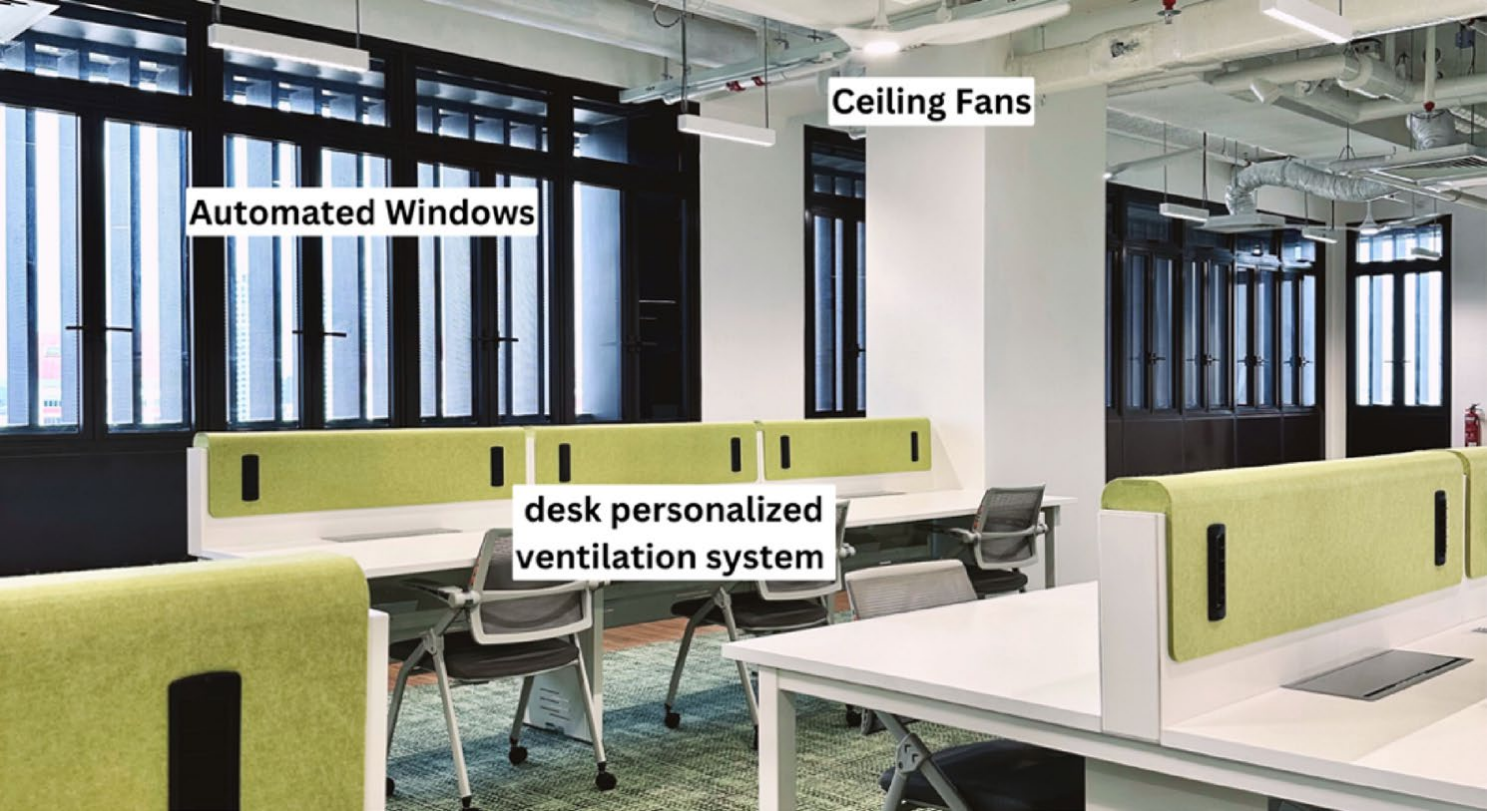
Illustration of Mixed-Mode Ventilation (MMV) System. *Note*. The MMV system integrates natural ventilation through automated operable windows, ceiling fans and a desk-based personalized ventilation system for localized cooling.

**Fig. 2:**
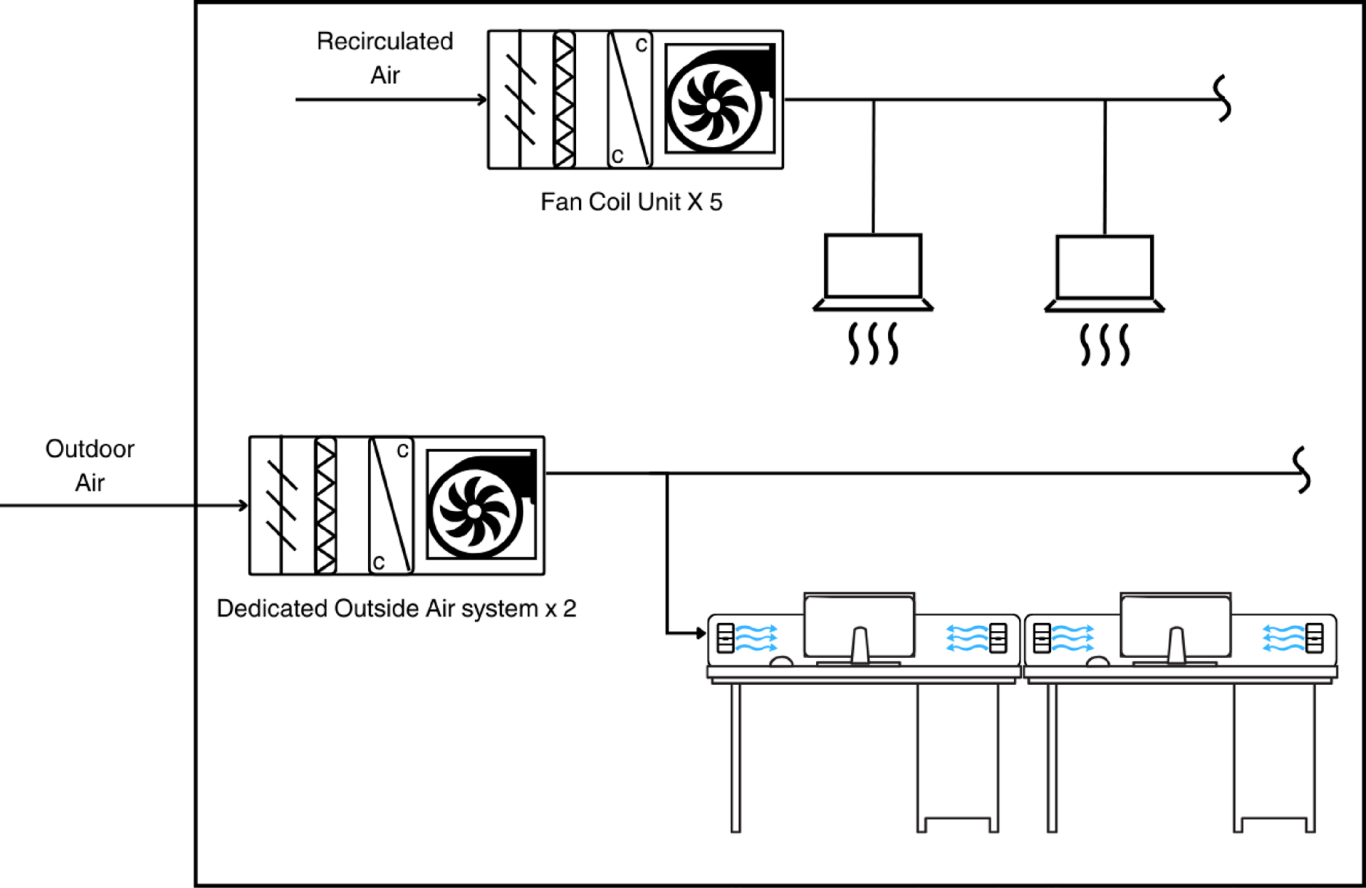
Diagram of the Air-Conditioning and Mechanical Ventilation (ACMV) System. *Note*. The ACMV system comprises two parts. One part is the fan coil unit (FCU) system that aims to provide mechanical cooling during AC operation. The other part is the dedicated outside air system (DOAS), designed and installed as a desk-level personalized cooling system. The DOAS serves two purposes. One is to provide mandatory ventilation during the AC operation when windows are closed. The second purpose is to provide intermediate localized cooling during the concurrent cooling (CC) mode during Study 1.

Employing both an experience sampling study and a randomized controlled trial, this research shows the effectiveness of a potential solution in addressing climate change while still providing an optimal work environment to employees. In doing so, this study contributes to existing scholarship in several ways. First, few studies have studied the impact of the use of ACs in environmental and organizational psychology. This may be because ACs are not considered as necessities in most WEIRD societies^15^. In non-WEIRD societies that tend to experience higher temperatures throughout the year, most organizations rely on ACs to maintain a conducive environment for employee well-being and productivity. This work adopts an interdisciplinary approach to study a potential solution that can address both the issue of environmental impact and organizational outcomes. Second, past studies on MMV have mainly focused on the energy performance of the buildings using MMV and the occupants’ perceived thermal comfort^14^. This paper studies this technology in the organizational context to examine how employees react to this system psychologically and behaviorally, and how the use of MMV may affect employees’ job satisfaction, work engagement, and job performance, which are important organizational goals. If the findings show no significant differences in work outcomes between AC and MMV settings, this will uncover a powerful way to reduce organizational energy use without sacrificing employee well-being or productivity, as the MMV system is a scalable design to replace the approximately two billion ACs that are currently in operation worldwide^16^.

Third, past studies on MMV have been conducted in countries with mild climates or with larger temperature ranges^17–20^. Thus, the MMV strategies employed in these past studies are not directly comparable to the MMV system employed in this current study, which takes place in a tropical climate. Consequently, this MMV system switches between natural ventilation and AC within the same day unlike what was employed in past studies. In addition, this system transitions at a much higher temperature threshold by leveraging elevated air movement from ceiling fans and localized cooling via a desk cooling system. Both of these factors are core enablers of the energy savings that can be achieved, and they reflect a fundamentally different MMV approach adapted for the tropical climate. Details regarding the MMV technology and design can be found in Appendix A, Figs. 1, 2, and S1. Thus, this paper aims to contribute to the literature by examining the use of a different MMV system in a non-WEIRD country^15^, Singapore, which has a tropical climate, characterized by hot and humid conditions. This paper focuses on studying the effects of the MMV system in the warm seasons, because past studies in the literature have mostly focused on colder climates, with only 9% of the case studies on MMV systems being conducted in tropical climates^21^. This is despite about 40% of the world’s population currently living in tropical climates, with a projected 50% of the global population living in tropical climates by 2050^22^. Thus, this provides a valuable opportunity to study how the MMV system works in warmer tropical climates with higher humidity. In addition, since the MMV system this paper studies adopts a more advanced multi-stage increment cooling approach, it can encourage more uptake of the MMV system in tropical climates if the studies can show their effectiveness in providing thermal comfort while reducing energy expenditure.

Fourth, the current literature on temperature and work performance remains inconclusive. A past meta-analysis shows that there is no relationship between temperature and work performance for a wide range of temperatures from 18 to 34 °C^23^, while a more recent meta-analysis shows that there is no significant difference in work performance when working under ideal temperature conditions of 21–25 °C compared to working under moderately reduced temperatures below 21 °C, but work performance decreased when working under moderately raised temperatures above 25 °C^24^. Due to the inconsistencies in these past findings and the relative lack of studies on the use of the MMV system in a tropical climate, it is important to assess the work-related outcomes of the MMV system adapted for tropical climates, where yearly average temperatures are already well above the upper range of the ideal temperature conditions of 25 °C.

## Results

### Study 1 results

Descriptive statistics and correlations among variables are displayed in Table S1.

**Fig. 3:**
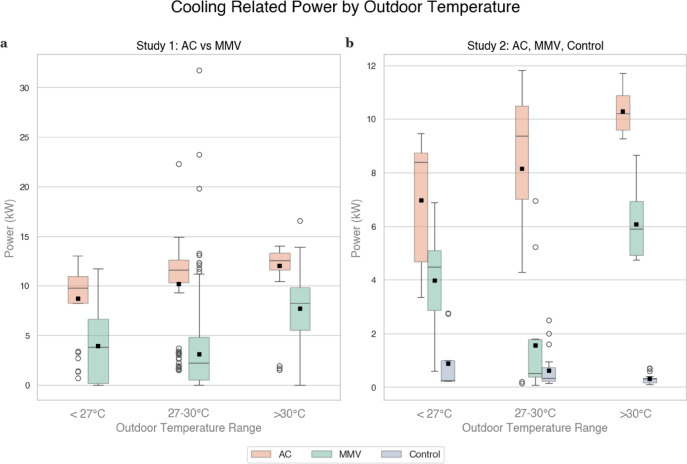
Studies 1 and 2 Cooling Power Comparison. *Note*. The white circles outside the box plots represent outliers. (**a**) In Study 1, the traditional AC operation generally exhibited the highest energy demand across all three outdoor temperature bins. During transitions from localized cooling to AC mode in the outdoor temperature range, MMV may yield a few spikes. When the temperature became more extreme (> 30 °C), MMV still exhibited lower power draw than traditional AC given its elevated cooling set point. (**b**) In Study 2, AC consistently showed the highest cooling‐related power. The NV control group remained near zero throughout, reflecting the shutdown of air conditioning and mechanical ventilation systems and ceiling fans. It should be noted that MMV exhibited higher power demand during favorable ambient conditions (< 27 °C) compared to moderate conditions (27–30 °C), because during one session (October 6^th^) of the MMV experimental condition rainfall triggered automatic window closure and temporarily forced the system to run in full AC mode, thereby increasing cooling energy use.

To compare the energy usage between an MMV and an AC system, Analysis of Covariance (ANCOVA) was conducted to examine differences in cooling power demands between the MMV and AC operations. ANCOVA (using Type III sums of squares to accommodate unbalanced operation group sizes) helps partial out the continuous effect of outdoor air temperature and isolate the effect of system type on energy savings. Results showed that types of system operation,* F*(1, 1151) = 459.38, *p* < 0.001, ${\eta }_{p}^{2}$ = 0.29, and outdoor air temperature, *F*(1, 1151) = 422.28, *p* < 0.001, ${\eta }_{p}^{2}$ = 0.27, are significant, suggesting that the MMV system consistently uses less cooling power than traditional AC systems, even when accounting for variances across different experiment periods in outdoor temperature. Fig. 3 and Table 1 illustrate the cooling power demand reduction of MMV compared to AC across different outdoor temperature ranges (< 27 °C, 27–30 °C, and > 30 °C).

**Table 1:** Energy-Saving Effect of the MMV System.

Outdoor Temperature Range	AC Mean Power (kW)	MMV Mean Power (kW)	Absolute Savings (kW)	Percent Reduction
*Study 1*				
< 27 °C	8.7	3.9	4.8	55
27–30 °C	10.2	3.1	7.1	70
> 30 °C	12.0	7.7	4.3	36
*Study 2*				
< 27 °C	7.0	4.0	3.0	43
27–30 °C	8.1	1.6	6.5	80
> 30 °C	10.2	6.0	4.2	41

*Note*. The reason why the MMV power when outdoor temperature is less than 27 °C is higher than that in the range of 27–30 °C is that there was a rain condition during the morning session on October 6^th^ when the outdoor temperature was low, but the windows were fully closed and AC mode was activated since it was raining outside per MMV rules.

To examine if there are any significant differences in work outcomes between Surveys 1.1 and 1.2 (traditional AC period), Surveys 2 to 10 (treatment period), and Survey 11 (traditional AC period), a repeated-measures analysis was conducted with the experimental condition (AC vs. MMV) as the within-subject variable, time as the continuous independent variable, and job satisfaction as the dependent variable. Results showed that the main effect of time on job satisfaction was not significant, *F*(11, 44) = 0.84, *p* = 0.60, ${\eta }_{p}^{2}$ = 0.17, suggesting that across time, changing from the traditional AC condition to the MMV condition and back to the traditional AC condition did not have a significant effect on employees’ job satisfaction. This result was the same for the other dependent variables as well, as shown here. The main effect of time on work engagement was not significant, *F*(11, 44) = 1.35, *p* = 0.23, ${\eta }_{p}^{2}$ = 0.25. This suggests that work engagement was not significantly different for employees when working under the AC and MMV conditions. The main effect of time on supervisor-rated job performance was also not significant, *F*(11, 22) = 0.55, *p* = 0.84, ${\eta }_{p}^{2}$ = 0.22. This suggests that job performance was not significantly different for employees when working under the AC and MMV conditions.

Exploratory data on helping behaviors were also collected. The main effect of time on helping behaviors was likewise not significant, *F*(11, 44) = 0.85, *p* = 0.59, ${\eta }_{p}^{2}$ = 0.17, suggesting that across time, there was no significant difference in employees’ helping behaviors when working under AC and MMV conditions.

Since the AC and MMV conditions were implemented at different times, the outdoor air temperature during the study period was recorded to evaluate if any differences in the outdoor air temperature during the study period may impact the interpretation of the results. The data on the outdoor air temperature during the different conditions are reported in Table 2. The MMV condition period had a mean outdoor air temperature of 29.83 °C compared to 29.11 °C during the AC condition period, with a difference of 0.72 °C. Despite facing more challenging thermal conditions (i.e., higher mean outdoor air temperature), the MMV system still achieved significant cooling power reductions, as shown in Fig. 3 and Table 1. This finding strengthens the conclusion that the energy savings resulted from the intelligent control strategy of the MMV system rather than favorable weather. ANCOVA further confirms this by explicitly controlling for outdoor temperature as a covariate (*F*(1, 1151) = 459.38, *p* < 0.001, ${\eta }_{p}^{2}$ = 0.29), ensuring that the significant effect of system type (*F*(1, 1151) = 422.28, *p* < 0.001, ${\eta }_{p}^{2}$ = 0.27) is independent of meteorological variations.

**Table 2:** Comparison of Outdoor Air Temperature by Conditions in Studies 1 and 2.

Condition	Hours	Mean Outdoor Air Temperature	Median Outdoor Air Temperature
*Study 1*			
AC	174	29.108908	29.075
MMV	980	29.8337628	30.0481061
*Study 2*			
AC	30	28.5109444	28.8725
MMV	26	28.8615385	28.9408333
Control (NV)	48	28.9864583	29.0275

*Note*. The difference in the number of hours for the 3 conditions in Study 2 was because some sessions had more participant sign-ups than others, resulting in a faster data collection for some conditions than for others.

### Study 2 results

**Fig. 4:**
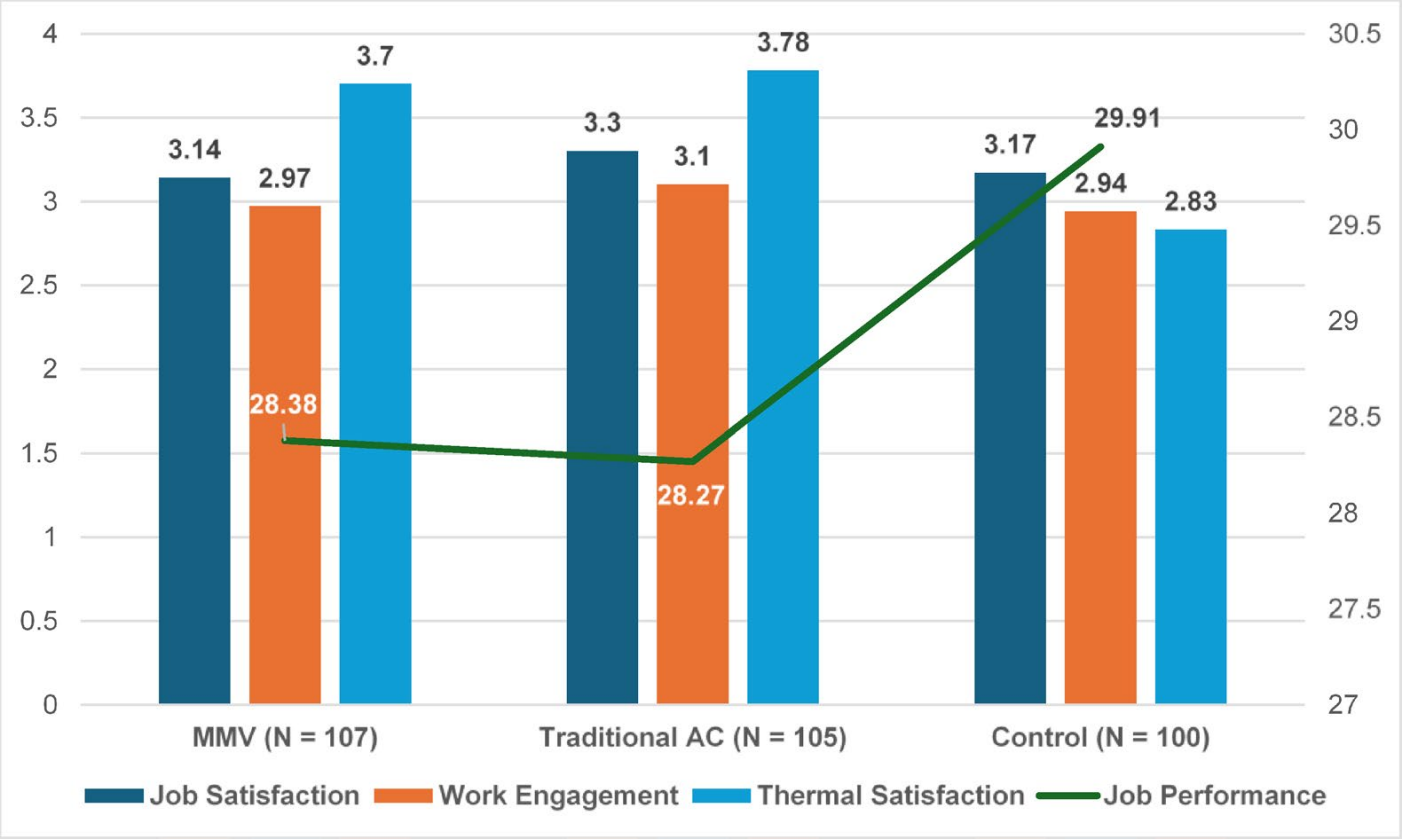
Graphs of Descriptive Statistics by Condition (Study 2). *Note*. Job satisfaction, work engagement, and thermal satisfaction are reported as bar charts following the left axis. Job performance is reported as a line graph following the right axis.

Overall descriptive statistics and correlations among variables are displayed in Table S2. Descriptive statistics by condition are displayed in Fig. 4 and Table 3.

**Table 3:** Descriptive Statistics by Condition and Post-hoc Analysis Results (Study 2).

Variables/Condition	MMV (*N* = 107)	Traditional AC (*N* = 105)	Control (*N* = 100)
Job Satisfaction	3.14 (0.90)	3.30 (0.75)	3.17 (0.75)
Work Engagement	2.97 (1.02)	3.10 (0.95)	2.94 (0.99)
Job Performance	28.38 (6.69)	28.27 (6.52)	29.91 (7.06)
Thermal Satisfaction	3.70 (1.02)^a^	3.78 (1.07)^b^	2.83 (1.17)^a, b^

*Note*. Standard deviations are given in parentheses. Job performance measure is a total aggregated score across the five tasks, with a full score out of 50. The superscripts a and b represent a significant difference at *p* < 0.05 between the MMV and control conditions, and between the traditional AC and control conditions respectively.

The energy usage between an MMV and an AC system was compared using ANCOVA to control for the effect of outdoor air temperature. The data showed that the system operation type (MMV vs. AC vs. Control) is the dominant predictor of cooling‐related power, *F*(2, 100) = 123.61, *p* < 0.001, ${\eta }_{p}^{2}$ = 0.71. Although outdoor air temperature was also significant, *F*(1, 100) = 5.08, *p* = 0.026, ${\eta }_{p}^{2}$ = 0.05, its effect was much smaller than that of system operation type. Fig. 3 and Table 1 align with the ANCOVA results, demonstrating the energy savings of MMV compared to AC across different outdoor temperature ranges.

The effects of the experimental conditions (MMV vs. AC vs. Control) on the thermal comfort and three work-related variables were also examined by conducting multivariate analysis of variance (MANOVA). MANOVA was chosen since it accounts for potential intercorrelations among the dependent variables. There is a significant difference across the three experimental conditions on the four combined dependent variables, *Wilks’ Lambda* = 0.85, *F*(8, 612) = 1.24, *p* < 0.001, ${\eta }_{p}^{2}$ = 0.08. This indicates that the experimental conditions had a significant effect on the four dependent variables collectively. Univariate tests revealed a significant difference among experimental conditions for thermal comfort, *F*(2, 309) = 23.893, *p* < 0.001, ${\eta }_{p}^{2}$ = 0.13. There were no significant differences among experimental conditions for job satisfaction, *F*(2, 309) = 1.11, *p* = 0.33, ${\eta }_{p}^{2}$ = 0.01; work engagement, *F*(2, 309) = 0.80, *p* = 0.45, ${\eta }_{p}^{2}$ = 0.01; and job performance, *F*(2, 309) = 1.87, *p* = 0.16, ${\eta }_{p}^{2}$ = 0.01.

Post hoc comparisons using the Bonferroni test indicated that participants in the MMV condition reported no significant differences in thermal comfort (*M* = 3.70, *SD* = 1.02) compared to those in the traditional AC condition (*M* = 3.78, *SD* = 1.07, *p* = 1.00). Post hoc comparisons using the Bonferroni test indicated that participants in the MMV condition reported significantly higher thermal comfort (*M* = 3.70, *SD* = 1.02) compared to those in the control condition (*M* = 2.83, *SD* = 1.17, *p* < 0.001).

Post hoc comparisons using the Bonferroni test indicated that participants in the MMV condition reported no significant differences in job satisfaction (*M* = 3.14, *SD* = 0.90) compared to those in the traditional AC condition (*M* = 3.30, *SD* = 0.75, *p* = 0.47), no significant differences in work engagement (*M* = 2.97, *SD* = 1.02) compared to those in the traditional AC condition (*M* = 3.10, *SD* = 0.95, *p* = 0.98), and no significant differences in job performance (*M* = 28.38, *SD* = 6.69) compared to those in the traditional AC condition (*M* = 28.27, *SD* = 6.52, *p* = 1.00).

Post hoc comparisons using the Bonferroni test indicated that participants in the MMV condition reported no significant differences in job satisfaction (*M* = 3.14, *SD* = 0.90) compared to those in the control condition (*M* = 3.17, *SD* = 0.75, *p* = 1.00), no significant differences in work engagement (*M* = 2.97, *SD* = 1.02) compared to those in the control condition (*M* = 2.94, *SD* = 0.99, *p* = 1.00), and no significant differences in job performance (*M* = 28.38, *SD* = 6.69) compared to those in the control condition (*M* = 29.91, *SD* = 7.06, *p* = 0.32).

Since the AC and MMV conditions were implemented at different times, the outdoor air temperature during the study period was recorded to evaluate if any differences in the outdoor air temperature during the study period may impact the interpretation of the results. The data on the outdoor air temperature during the different conditions are reported in Table 2. The MMV condition period experienced a mean outdoor air temperature of 28.86 °C compared to 28.51 °C during the AC condition period, with a difference of 0.35 °C. The mean outdoor air temperature during the control condition was 28.99 °C.

## Discussion

With a 19-week experience sampling field study design and a field experiment with random assignment, the studies tested the efficacy of MMV system in providing occupants with thermal comfort while still maintaining a similar level of job performance, job satisfaction, and work engagement as traditional ACs. Given the crucial and urgent nature of climate change, if the MMV system is found to be effective in providing thermal comfort and producing similarly positive work outcomes as using traditional ACs, this could potentially be a solution that organizations can start implementing to achieve their environmental sustainability goals without affecting the productivity of their employees. The studies provide initial evidence that there were no statistically significant differences in the participants’ job satisfaction, work engagement, and job performance between those working under the MMV condition and those working under traditional AC condition, as reported in the Results section. The data also showed that the MMV system had significant effects on the thermal comfort level of occupants when compared to the no-intervention control group, highlighting both the thermal discomfort usually felt in tropical climates and the ability of the MMV system to overcome that by providing a similar level of thermal comfort to the traditional AC condition (Table 3). Thus, this research reveals a potentially scalable intervention that can reduce climate change without sacrificing workplace productivity and well-being.

There are strengths and limitations of the empirical approach that are worth noting in interpreting the results of these studies. One strength is that this paper used both a field study design and an experimental study design that compares three conditions. This provides both external validity to generalize the findings to real world situations across different organizations, thus increasing its practical implications, and internal validity to make causal inferences. However, one limitation is the small sample size of Study 1 due to the relatively small headcount of the technology company that was currently renting the office, which might limit the generalizability of the findings. In addition, the low statistical power from the small sample size may render the non-significant findings uninterpretable, as Study 1 is highly susceptible to Type II errors, where a true effect may fail to be detected. Due to the longitudinal nature of Study 1, and the need for prolonged usage of the office space in order to conduct Study 1, it was not possible to recruit more external participants outside of the technology company that was currently renting the office, resulting in the small sample size. Nevertheless, the effects of a small sample size in the number of participants (*N* = 19) are partially mitigated by the larger sample size in terms of data points (*N* = 168). Since the purpose of Study 1 is to examine the within-person differences between working under the MMV condition versus the traditional AC condition, we focused more on collecting many repeated measures from the participants. What matters most for detecting within-person change over time is having many repeated measurements per individual (level 1 occasions), because a larger number of waves increases precision in estimating trajectories^25^. This is also another strength of the Study 1 research design, which is that it was conducted over a relatively long period of time of 19 weeks, with bi-weekly contact with the participants, which allowed for longitudinal data collection to study the long-term effects of working under the MMV system.

In addition to the limitation of a small sample size in Study 1, Study 1 participants were all employees of an organization that had already agreed to work in the office space with the MMV system installed. Thus, their perceived acceptability of the MMV system may be higher than the general population. Another limitation of Study 1 is that during the time of participation, the participants were already aware of the purpose of the study and were aware of the periods during which they would be subject to the AC and MMV conditions. This implies that the participants could have deliberately chosen different clothes to wear depending on the condition they would be subject to. However, since the participants were employees working in the office space when Study 1 was conducted, they were all wearing typical office wear to work under both the MMV and AC conditions. Nevertheless, these two limitations are partially addressed by Study 2, which employed a randomized controlled trial with a large sample of participants. Another potential limitation of the study package is the use of the same study site for both Studies 1 and 2, which might have resulted in limited generalizability of how MMV systems work across different types of buildings. This is because at the time of the study, the study site used is the only place in Singapore that has the personalized cooling system installed at each work station.

Another strength of the study design is the use of more objective measures of job performance compared to using a self-report measure, by obtaining supervisor-rated job performance data in Study 1 and actual job performance data in Study 2. Self-report measures have the potential to be less accurate than other-report measures, due to social desirability bias or other factors^26^. Especially for performance outcomes, it is likely to be less objective to receive ratings from the employees themselves.

A point worth noting is that no significant differences were found between participants’ job performance, job satisfaction, and work engagement when working under the MMV system compared to the no-intervention condition in Study 2, despite the significantly lower thermal comfort experienced by those in the no-intervention condition than those in the MMV condition (Table 3). However, this result should not be taken as evidence against past findings showing the importance of thermal environments and thermal comfort in affecting work outcomes^27–31^, because Study 2 was only a two-hour study that asked participants to complete standardized tasks. It is reasonable that participants would still be able to perform well and feel satisfied and engaged despite experiencing thermal discomfort. In contrast, if full-time employees have to work on more complex tasks under the no-intervention condition daily, then they may likely not be able to perform as well as they can in a comfortable environment. Although studies of a shorter duration are more feasible and less costly to conduct, future research should seek to conduct a full-day or multiple-day field experiment to fully examine if the lack of significant differences in job performance, job satisfaction, and work engagement when working under the MMV system compared to the no-intervention condition is due to the short duration of the study. Future research can also delve deeper into this line of research by examining other important workplace outcomes when working under the MMV system, and how the MMV system can be further enhanced to improve employee outcomes.

It has been well-documented in past research that heat can have physiological effects on the human body^32,33^. Thus, future researchers should also consider utilizing more objective physiological measures of well-being and thermal comfort, such as by measuring the participants’ heart rate, heart rate variability, and body temperature using wearable devices. This will provide more objective data on thermal comfort than just relying on self-report measures of thermal comfort.

In summary, the studies found that the MMV system is not only able to provide thermal comfort to occupants, but there were also no statistically significant differences found between occupants’ job performance, job satisfaction, and work engagement when they worked under the MMV system as when they worked under the traditional AC system. This could be a potential useful solution for organizations that aim to be environmentally sustainable. By implementing the MMV system in their physical workspaces, organizations may potentially be able to reap the environmental and financial benefits of lower energy consumption, while not sacrificing the productivity and job satisfaction of their employees.

## Methods

### Overview of studies

Two studies were conducted. To examine the long-term effects of working under the MMV system, Study 1, a 19-week experience sampling method (ESM) study that spanned from April to August 2024 with employees in an organization in Singapore, was conducted. To document causal effects, Study 2, a two-hour experiment among students that spanned from September to October 2024, was then conducted. The context in both studies was an 8073 square feet (or 750 square meter) commercial open-plan office space in Singapore, which is a living laboratory to evaluate MMV cooling approaches, with an MMV system installed. The office is equipped with desk-level personalized ventilation provided by dedicated outside air systems (DOAS), fan coil units (FCU) for mechanical cooling, and automated motorized windows. Details regarding its technology and design can be found in Appendix A, Figs. 1, 2, and S1.

### Details about the MMV system operation and the conditions employed in the studies

For both Studies 1 and 2, the system could be running in Natural Ventilation (NV) condition (only included in Study 2), AC condition, and MMV condition. The operating hours of the MMV or AC systems during Study 1 was 7.30am to 7 pm every weekday. Study 1 ran from 8 April 2024 to 18 August 2024 in Singapore. Study 2 was conducted in four two-hour study sessions each day over four weekends (9–11am; 11am–1 pm; 2–4 pm; 4–6 pm). Study 2 ran from 28 September 2024 to 20 October 2024 in Singapore.

The NV condition was included as a control condition in Study 2. Study 2 followed the convention in the literature on MMV studies on the use of the NV condition to compare against the AC and the MMV conditions^34–36^. Thus, the NV condition was included as a control condition to highlight the effects of temperature and thermal comfort on work-related outcomes when there is no intervention to provide thermal comfort to occupants.

The NV condition means that the windows were regulated to be open no matter what the outdoor air temperature was, with the ceiling fans switched off. The AC condition means that the windows were fully closed, and FCUs and DOAS were running concurrently to provide mechanical ventilation and mechanical cooling. The office space maintained a constant indoor air temperature set point at 24 °C and relative humidity of 60%, denoting a typical air-conditioned office setting in Singapore.

The MMV condition had been set to operate in different modes depending on the outdoor air temperature and weather condition. During Study 1, the MMV condition was running as follows. It runs in the NV mode when the outdoor air temperature is less than 29 °C and the weather is favorable. In the NV mode, the windows are regulated to open when the outdoor air temperature is lower than 29 °C and there are no rain, strong wind, or high pollution events outside. The system prioritizes natural airflow when conditions are favorable, considering factors such as wind speed, direction, and precipitation. The MMV condition runs in the Concurrent Cooling (CC) mode when the outdoor air temperature is between 29 and 31 °C and the weather is favorable. In the CC mode, the DOAS provides personalized cooling to occupants while maintaining open windows. The MMV condition runs in the AC mode when the outdoor air temperature is higher than 31 °C and/or during adverse weather. In the AC mode, the windows are fully closed, and the system switches entirely to mechanical cooling through FCUs and DOAS units. This mode is also activated during rain, haze, or high pollution events to maintain indoor air quality. A slightly elevated indoor temperature of 27 °C is maintained with supplementary ceiling fan operation to conserve energy and minimize abrupt thermal transitions between modes.

During Study 2, the MMV condition was running as follows. It runs in the NV mode when the outdoor air temperature is lower than 30 °C and the weather is favorable. In the NV mode, the windows are regulated to open when the outdoor temperature is lower than 30 °C and there are no rain, strong wind, or high pollution events outside. The system prioritizes natural airflow when conditions are favorable, considering factors such as wind speed, direction, and precipitation. The MMV condition runs in the AC mode when the outdoor air temperature is higher than 30 °C or during adverse weather. In the AC mode, the windows are fully closed, and the system switches entirely to mechanical cooling through FCUs and DOAS units. This mode is also activated during rain, haze, or high pollution events to maintain indoor air quality. A slightly elevated indoor air temperature of 27 °C is maintained with supplementary ceiling fan operation to conserve energy and minimize abrupt thermal transitions between modes.

Different MMV operation modes were used for Studies 1 and 2 due to their distinct research objectives and experimental designs. Study 1 is a longitudinal study that tests the efficacy of the MMV system in providing thermal comfort in the tropics over a longer period. As such, the MMV operation modes were designed using an innovative three-step incremental cooling approach, including the CC mode with desktop personalized ventilation systems. The purpose is two-fold. First, the CC mode is particularly valuable in the tropics, where extending the window opening period and delaying the AC mode yields substantial cumulative energy reductions. Second, the CC mode facilitates more gradual thermal stimulation, which is beneficial to the thermal adaptation of the participants. In contrast, Study 2 used a traditional NV and AC configuration without the intermediate CC mode. For a short-term cognitive performance study involving only a two-hour session, this setup ensured participants could experience clearly distinguishable thermal transitions within the limited timeframe, providing comprehensive immersion in the MMV operation. Including the CC mode in such short sessions would have reduced the contrast between conditions, introduced additional control complexity at the desk level, and, more importantly, would not have provided sufficient exposure time for the thermal adaptation processes that the CC mode is designed to leverage. The following references provide further details on MMV system and thermal conditions^37,38^.

### Study 1

Study 1 has received ethics approval from the National University of Singapore (ID: NUS-IRB-2023–97). Study 1 was performed in accordance with relevant guidelines and regulations. Informed consent was obtained from all participants.

#### Sample and procedure

**Fig. 5:**
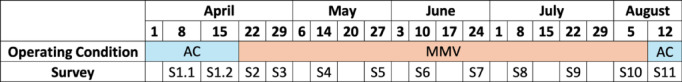
Study 1 Survey Timings. *Note*. For Study 1, in the first 2 weeks (April 8–April 21) and in the last 1 week (August 12–August 18), the participants used traditional AC in their office, and in the 16 weeks of treatment period (April 22–August 11), the participants used the MMV system in their office.

The office is currently rented by a technology company, with a total of 19 participants (14 employees, four interns, and one supervisor) agreeing to participate in the study. A 19-week ESM study was conducted, consisting of three phases: a 2-week pre-treatment period, a 16-week intervention period, and a 1-week post-treatment period. There was bi-weekly contact with participants (Fig. 5), during which employees reported on job satisfaction, work engagement, and helping behavior, and the supervisor rated each employee’s job performance. Data were collected twice during the 2-week pre-treatment period, nine times during the 16-week intervention period, and once during the 1-week post-treatment period, resulting in 12 data collection timepoints, as shown in Fig. 5. Two of the 14 employees reported to a different supervisor who did not participate, and the supervisor in this study reported to another manager who also declined participation. Therefore, there were only supervisor-rated job performance data for 16 out of 19 participants. Additionally, power meters were installed throughout the office space to measure energy expenditure during the study period.

For this final sample of 19 participants, 168 responses were collected out of a total possible 228 responses (= 19 participants * 12 data collection time points; 73.7% response rate at level 1). The mean age of the participants was 33.0 years (*SD* = 10.5), and 57.9% were male, with 89.5% being ethnic Chinese. The 19-week participation period was segmented into five waves, and the participants were reimbursed approximately USD 277 for taking part in all five waves.

#### Study 1 measures

*Job Satisfaction* Job satisfaction was measured using a 5-item job satisfaction scale^39^. A sample item is “*At this very moment, I feel fairly satisfied with my job*” (1 = Strongly disagree, 5 = Strongly agree) (α = 0.77).

*Work Engagement* Work engagement was measured using the 5-item work engagement scale^40^. A sample item is “*I am enthusiastic about my work*” (1 = Does not apply at all, 5 = Fully applies) (α = 0.98).

*Helping Behaviors* Helping behavior was measured using the 5-item altruism subscale of the organizational citizenship behavior measure^41^. A sample item is “*I help others who have heavy work loads*” (1 = Strongly disagree, 5 = Strongly agree) (α = 0.93).

*Supervisor-Rated Job Performance* Job performance was measured using the 4-item job performance scale^42^, rated by the participants’ supervisor. A sample item is “*Compared to his/her peers, (this employee) is an excellent worker*” (1 = Strongly disagree, 5 = Strongly agree) (α = 0.94).

*Demographic Variables* The participants were asked to indicate their age, gender, and race.

### Study 2

Study 2 has received ethics approval from the National University of Singapore Business School (ID: MNO-24–0725). Study 2 was performed in accordance with relevant guidelines and regulations. Informed consent was obtained from all participants.

#### Sample and procedure

Study 1 provided correlational evidence of the effectiveness of the MMV system in terms of energy savings and employee outcomes, but its design precludes causal inferences. Therefore, an experiment was conducted with participants in the same office space over weekends, because the tenants occupied the office space throughout weekdays. To avoid drawing participants’ attention to the environmental condition, an element of deception was employed in this study, by informing participants that the aim of the study was to test the effect of task completion on job satisfaction and work engagement. Due to the deception employed, a debrief was provided to the participants on the true nature and aim of the study at the end and they were given the option to withdraw their data, in line with the ethics approval guidelines. Participants included undergraduate and postgraduate students as well as staff of a Singapore university affiliated with the authors. However, since no further questions were asked on the participants’ identity after checking for their affiliation with the university, it is not possible to provide a breakdown of the number of each type of participants who took part in Study 2. Participants were given the option to be reimbursed with either cash (approximately USD 29) or course credits for those recruited from the undergraduate subject pool.

The aim of Study 2 was to collect at least 100 usable responses for each of the three conditions: 1) traditional AC (operated at 24 °C without ceiling fans), 2) MMV system, and 3) control (NV condition, which is at room temperature without traditional AC, MMV system, or ceiling fans). The participants were invited to the office for a two-hour study and randomly assigned into one of the three experimental conditions using a block randomization method, assigning a different condition per day. However, due to the agreement with the building tenants on the use of the office space on weekends for the study, Study 2 had to be completed within 4 weekends, which resulted in the last day of data collection being the AC condition from 9 to 11am and the control condition for the remaining 3 sessions, in order to ensure there were 100 usable responses for each condition. Four two-hour study sessions were conducted each day over four weekends (9–11am; 11am–1 pm; 2–4 pm; 4–6 pm). The participants were asked to complete a series of tasks measuring work performance, including typing, numerical calculation, memory-recall, anagram-solving, and creativity, and at the end of these tasks, they reported on job satisfaction^39^ and work engagement^40^, as well as other measures on thermal comfort. To prevent the demand effect from asking questions related to thermal comfort, the thermal comfort measure was included right at the end of the study after all other measures were completed by the participants. Details of the tasks and the items are in Appendix B. Participants received five-minute breaks between each task. The tasks below were ordered such that all participants began with the typing task and finished with the creativity task, with the four tasks in between randomized in their order of appearance for all participants.

A total of 359 participants took part in this study; 12 participants indicated they would like to withdraw their data; 8 participants faced technical difficulties such as the Qualtrics survey being timed out or not being able to submit their Qualtrics survey; 17 participants did not take part in the study under their pre-assigned experimental conditions due to an administrative error. Finally, 10 additional participants’ data were discarded because a fire alarm went off during their study session due to a small flame in a nearby building, which resulted in a disrupted study session. The final sample consisted of 312 participants, with 107 participants in the MMV condition, 105 participants in the traditional AC condition, and 100 participants in the control condition. The mean age of the participants was 22.0 years (*SD* = 2.8). 56.1% were female, with 70.5% being ethnic Chinese.

#### Study 2 tasks

*Typing Task* At the start of the session, participants were asked to complete 10 min of typing task on a typing practice website (https://monkeytype.com) at their own pace. Afterwards, participants were asked to self-report their typing speed in words per minute, accuracy rate, and consistency rate, all of which were provided by the website at the end of the typing task. This task is a filler task to get the participants acclimatized to the thermal environment, and the performance measures for this task were not used in the calculation of the overall job performance score.

*Addition Task* Participants were given 20 columns of randomly generated five two-digit numbers. They were asked to add as many columns as possible in 10 min (adapted from Wargocki et al.^43^).

*Multiplication Task* Participants were given 20 columns of randomly generated two two-digit numbers. They were asked to multiply each pair (adapted from Lan et al.^44^), and asked to complete as many columns as possible in 10 min.

*Memory-Recall Task* Participants were given ten minutes to memorize a list of 15 “paired-associate” words randomly chosen from Underwood’s^45^ list of 200 paired-associate words. They were then presented with a list of words from one word of each of the 15 pairs, and given five minutes to recall the other word. This followed the precedent of the memory recall task used^46^.

*Anagram Task* Participants were given instructions on how to solve anagrams, and given 15 min to solve 20 moderately difficult anagrams^47^.

*Creativity Task* After being informed that creative ideas consist of ideas that are novel and useful at the same time, participants were given five minutes to generate creative ideas for a decorative object to be sold in the university shop^48^.

#### Study 2 measures

*Job Performance* Job performance was measured based on the participants’ performance in the five tasks above, excluding the typing task. For the addition task, the number of accurate additions completed was used as a performance measure, with a full score of 20. For the multiplication task, the number of accurate multiplications completed was used as a performance measure, with a full score of 20. For the memory-recall task, the number of accurate words recalled was used as a performance measure, with a full score of 15. For the anagram task, the number of anagrams solved accurately was used as a performance measure, with a full score of 20. For the creativity task, participants generated 2,160 ideas in total. Similar to past research employing a crowdsourcing approach to creativity ratings^49–51^, 467 Prolific participants were recruited and asked to rate each idea using a three-item scale (i.e., This idea is novel/useful/creative; 1 = Strongly disagree to 7 = Strongly agree). All raters were blind to the experimental conditions, and each rated 50 ideas for creativity. On average, each idea was rated by 10.41 unique raters. The average score was calculated across these three items for all the ideas per participant and used as a performance measure for each participant (α = 0.85). Afterwards, all the scores across these five tasks were re-weighted such that each task has a full score of 10. The overall score across these five tasks, out of a full score of 50, was then obtained by summing the re-weighted scores to measure aggregate job performance.

*Job Satisfaction* Job satisfaction was measured using the 5-item job satisfaction scale^39^. A sample item is “*At this very moment, I feel fairly satisfied with my job*” (1 = Strongly disagree, 5 = Strongly agree) (α = 0.82).

*Work Engagement* Work engagement was measured using the 5-item work engagement scale^40^. A sample item is “*I am enthusiastic about my work*” (1 = Does not apply at all, 5 = Fully applies) (α = 0.93).

*Thermal Comfort* Based on the definition of thermal comfort being a state of satisfaction with the thermal environment^10^, thermal comfort was measured using a self-generated question, “*How satisfied are you with the overall thermal environment right now?*” (1 = Very dissatisfied, 5 = Very satisfied).

*Demographic Variables* Participants were asked to indicate their age, gender, and race.

## Supplementary Information

Below is the link to the electronic supplementary material.

Supplementary Material 1

## Data Availability

All data relevant to the study are included in the article or uploaded as supplementary information. In addition, all data, analysis codes, and materials are available on a dedicated Open Science Framework (OSF) webpage (https://osf.io/mf3cg/?view_only=c597b7a2664d4a96b32e11eff0e73c2a). Data were analyzed using Jupyter Notebook that uses Python programming language and SPSS version 29.0.0.0.
