# Supplementary material for: The effects of mixed-mode ventilation on energy saving and employee job satisfaction, work engagement, and job performance
Source: Sci Rep. 2026 Feb 12;16:8463. doi: 10.1038/s41598-026-38812-0 (PMC12972336; doi:10.1038/s41598-026-38812-0)
Supplement: Supplementary file 1 — Supplementary Material 1 [file 41598_2026_38812_MOESM1_ESM.docx]

**The Effects of Mixed-Mode Ventilation on Energy Saving and Employee Job Satisfaction, Work Engagement, and Job Performance**

Yamon Min Ye^1*^, Wei Liang^2^, Fei Xu^1^, Adrian Chong^2^, Christopher M. Barnes^3^, & Kai Chi Yam^1^

Department of Management and Organisation^1^

National University of Singapore

Department of the Built Environment^2^

National University of Singapore

Department of Management and Organization^3^

University of Washington

**Author Note**

Yamon Min Ye [
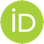
](https://orcid.org/0009-0001-2682-7626) https://orcid.org/0009-0001-2682-7626

Wei Liang [
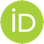
](https://orcid.org/0000-0002-4559-6982) https://orcid.org/0000-0002-4559-6982

Fei Xu [
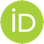
](https://orcid.org/0009-0009-5901-8487) https://orcid.org/0009-0009-5901-8487

Adrian Chong [
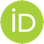
](http://orcid.org/0000-0002-9486-4728) http://orcid.org/0000-0002-9486-4728

Christopher Barnes [
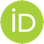
](https://orcid.org/0000-0003-2520-6200) https://orcid.org/0000-0003-2520-6200

Kai Chi Yam [
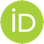
](http://orcid.org/0000-0001-7381-8039) http://orcid.org/0000-0001-7381-8039

Correspondence concerning this manuscript should be addressed to Yamon Min Ye (Email: [yamonminye@u.nus.edu](mailto:yamonminye@u.nus.edu))

**Appendix A: Technology and Design of the Mixed-Model Ventilation (MMV) System**

MMV combines natural ventilation from operable windows with mechanical air-conditioning to provide thermal comfort to occupants while reducing energy consumption. Although proven effective, MMV implementation has been limited in tropical climates due to preconceptions abouts its effectiveness under constant hot and humid conditions. For example, Chen *et al*.^1^ used simulations to conclude that countries in the tropics have little to no natural ventilation potential. However, the MMV system studied in this paper is uniquely designed not to rely solely on passive natural ventilation. First, it prioritizes using natural ventilation when outdoor air temperature is below 29°C, relying on ceiling fans for thermal comfort. As temperature rises, a desk-based personalized ventilation system gets activated. The objective is to cool the space around an occupant, avoiding the energy-intensive cooling of the entire space that traditional ACs do. Beyond 31°C, the windows automatically close, and the central cooling system is turned on. Notably, even in this mode, indoor temperatures are maintained at 27°C, higher than the conventional 23-25°C, utilizing air movement from the ceiling fans to continually ensure occupant thermal comfort and energy savings.

**Appendix B: Studies 1 & 2 Survey Items and Tasks**

**Study 1 Survey Items**

**Job Satisfaction**^2^ [1 = Strongly Disagree, 5 = Strongly Agree]

Please rate the extent to which you agree with each statement with regards to today.

1. Right now, I find real enjoyment in my work.
2. During most of the past hour I have felt enthusiastic about my work.
3. At this very moment, I feel fairly satisfied with my job.
4. Right now, each minute of work seems like it will never end. (Reverse-coded)
5. At the present time, I consider my job rather unpleasant. (Reverse-coded)

**Work Engagement**^3^ [1 = Does not apply at all, 5 = Fully applies]

Please rate the extent to which each statement applies to you with regards to today.

1. I feel strong and vigorous in my work.
2. At my work, I feel bursting with energy.
3. I am enthusiastic about my work.
4. My work inspires me.
5. I’m happily engrossed in my work.

**Helping Behaviors**^4^ [1 = Strongly Disagree, 5 = Strongly Agree]

Please rate the extent to which you agree with each statement with regards to today.

1. I help others who have been absent.
2. I help others who have heavy work loads.
3. I help orient new people even though it is not required.
4. I willingly help others who have work related problems.
5. I am always ready to lead a helping hand to those around me.

**Supervisor-Rated Job Performance**^5^ [1 = Strongly Disagree, 5 = Strongly Agree]

Please rate the extent to which you agree with each statement about (this employee) with regards to today.

1. All things considered, (this employee) is outstanding at his/her job.
2. Compared to his/her peers, (this employee) is an excellent worker.
3. (This employee) is one of the best at what he/she does.
4. (This employee) is very good at his/her daily job activities.

**Demographics**

Age, Gender, Race

**Study 2 Tasks**

**Typing Task (10 minutes)**

At the start of the session, participants were asked to complete 10 minutes of typing task on a typing practice website (<https://monkeytype.com>) at their own pace. Afterwards, the participants were asked to self-report their typing speed in words per minute, accuracy rate, and consistency rate, all of which were provided by the website at the end of the typing task. This task is a filler task to get the participants acclimatized to the thermal environment, and the performance measures for this task were not used in the calculation of the overall job performance score.

**< 5 minute break >**

**Addition Task (10 minutes)**

Participants were given 20 columns of randomly generated five two-digit numbers and were asked to add as many columns as possible in 10 minutes. This task is adapted from the addition task used in Wargocki *et al*.^6^. The number of accurate additions completed was used as a performance measure.

**< 5 minute break >**

**Multiplication Task (10 minutes)**

Participants were given 20 columns of randomly generated two two-digit numbers, and were asked to multiply as many columns as possible in 10 minutes. This task is adapted from the numerical calculation task used in Lan *et al*.^7^. The number of accurate multiplications completed was used as a performance measure.

**< 5 minute break >**

**Memory-Recall Task (15 minutes)**

Participants were given 10 minutes to memorize a list of 15 “paired-associate” words randomly chosen from Underwood’s^8^ list of 200 paired-associate words. The participants were then be presented with a list of words from one of each of the 15 paired-associate words and given five minutes to recall the other word, similar to the memory recall task used in Porath & Erez^9^. The number of accurate words recalled was used as a performance measure.

15 “paired-associate” words:

1. Crowd-photo
2. Usual-layer
3. Merge-guard
4. Taste-crypt
5. Spicy-heard
6. Guilt-fated
7. Mural-wiper
8. Fault-birth
9. Thumb-comic
10. Moody-greet
11. Daily-juror
12. Poser-comma
13. Sewer-forgo
14. Exude-skies
15. Acute-ultra

**< 5 minute break >**

**Anagram Task (15 minutes)**

The participants were given instructions on how to solve anagrams, and given 15 minutes to solve 20 anagrams that are moderate in difficulty (e.g., Erez & Isen^10^). The number of anagrams solved accurately was used as a performance measure.

Below is a list of 20 anagrams. To solve an anagram, you have to rearrange the letters of each of the words given to form a different word, using all the original letters exactly once. Please solve as many of them as you can within the next 20 minutes.

20 anagrams:

1. Handouts [Answer: Thousand]
2. Stream [Answer: Master]
3. Paired [Answer: Repaid]
4. Silence [Answer: License]
5. Deliver [Answer: Relived, Reviled]
6. Topside [Answer: Deposit]
7. Specter [Answer: Respect]
8. Notices [Answer: Section]
9. Chapter [Answer: Repatch]
10. Reactive [Answer: Creative]
11. Hornet [Answer: Throne]
12. Meteor [Answer: Remote]
13. Thread [Answer: Hatred, Dearth]
14. Breath [Answer: Bather]
15. Specie [Answer: Pieces]
16. Nation [Answer: Anoint]
17. Scramble [Answer: Clambers]
18. Friend [Answer: Finder]
19. Download [Answer: Woodland]
20. Terrain [Answer: Trainer, Retrain]

**< 5 minute break >**

**Creativity Task (5 minutes)**

Participants were given 5 minutes to generate creative ideas for a decorative object to be sold in the university shop^11^. Participants were informed that creative ideas consist of ideas that are novel and useful at the same time. Prolific participants were then asked to rate all the ideas generated using a three-item scale (i.e., This idea is novel/useful/creative; 1 = Strongly disagree to 7 = Strongly agree). The average score across these three items for all the ideas per participant was calculated and used as a performance measure for each participant.

**Study 2 Survey Items**

**Job Satisfaction**^2^ [1 = Strongly Disagree, 5 = Strongly Agree]

Please rate the extent to which you agree with each statement with regards to the tasks you have completed during this study.

1. Right now, I find real enjoyment in my work.
2. During most of the past hour I have felt enthusiastic about my work.
3. At this very moment, I feel fairly satisfied with my job.
4. Right now, each minute of work seems like it will never end. (Reverse-coded)
5. At the present time, I consider my job rather unpleasant. (Reverse-coded)

**Work Engagement**^3^ [1 = Does not apply at all, 5 = Fully applies]

Please rate the extent to which each statement applies to you with regards to the tasks you have completed during this study.

1. I feel strong and vigorous in my work.
2. At my work, I feel bursting with energy.
3. I am enthusiastic about my work.
4. My work inspires me.
5. I’m happily engrossed in my work.

**Thermal Comfort**

1. How satisfied are you with the overall thermal environment right now? [1 = Very dissatisfied, 5 = Very satisfied]

**Demographics**

Age, Gender, Race

**Figure S1**

*Floor Plan of Office Space*

*Note*. Locations of operable windows, ceiling fans, and desk personalized ventilation system are indicated in the figure.

**Table S1**

*Descriptive Statistics and Bivariate Correlations (Study 1)*

|  |  | M | SD | 1 | 2 | 3 | 4 | 5 | 6 | 7 |
| --- | --- | --- | --- | --- | --- | --- | --- | --- | --- | --- |
| 1 | Age | 34.19 | 10.69 | – |  |  |  |  |  |  |
| 2 | Gender | 0.55 | 0.50 | –0.01 | – |  |  |  |  |  |
| 3 | Race | 1.07 | 0.36 | –0.06 | –0.15^*^ | – |  |  |  |  |
| 4 | Job Satisfaction | 3.51 | 0.73 | –0.07 | –0.19^*^ | –0.07 | **0.77** |  |  |  |
| 5 | Work Engagement | 3.00 | 1.06 | –0.10 | –0.17^*^ | 0.17^*^ | 0.62^**^ | **0.98** |  |  |
| 6 | Job Performance | 3.57 | 0.74 | –0.07 | –0.34^**^ | –0.11 | 0.21^*^ | 0.21^*^ | **0.94** |  |
| 7 | Helping Behaviors | 3.76 | 0.91 | 0.19^*^ | –0.40^**^ | 0.21^**^ | 0.36^**^ | 0.40^**^ | 0.11 | **0.93** |

*Note*. *N* = 19. Gender is coded as 1 = Male, 0 = Female. Race is coded as 1 = Chinese, 2 = Indonesian, 3 = Burmese. Internal consistency reliabilities are shown on the diagonal in bold. ^*^ *p* < 0.05, ^**^ *p* < 0.01.

**Table S2**

*Overall Descriptive Statistics and Bivariate Correlations (Study 2)*

|  |  | M | SD | 1 | 2 | 3 | 4 | 5 | 6 | 7 |
| --- | --- | --- | --- | --- | --- | --- | --- | --- | --- | --- |
| 1 | Age | 21.97 | 2.77 | – |  |  |  |  |  |  |
| 2 | Gender | 1.58 | 0.52 | –0.22^**^ | – |  |  |  |  |  |
| 3 | Race | 1.66 | 1.06 | 0.05 | –0.03 | – |  |  |  |  |
| 4 | Job Satisfaction | 3.20 | 0.80 | 0.16^**^ | 0.11^*^ | 0.11 | **0.82** |  |  |  |
| 5 | Work Engagement | 3.00 | 0.99 | 0.07 | 0.05 | 0.13^*^ | 0.67^**^ | **0.93** |  |  |
| 6 | Job Performance | 28.83 | 6.77 | –0.02 | –0.02 | 0.13^*^ | 0.22^**^ | 0.20^**^ | – |  |
| 7 | Thermal Satisfaction | 3.45 | 1.17 | 0.11 | 0.05 | 0.04 | 0.20^**^ | 0.11^*^ | –0.05 | – |

*Note*. *N* = 312. Gender is coded as 1 = Male, 2 = Female, 3 = Non-binary/third gender, 4 = Prefer not to say. Race is coded as 1 = Chinese, 2 = Malay, 3 = Indian, 4 = Others. Job performance measure is a total aggregated score across the five tasks, with a full score out of 50. Internal consistency reliabilities are shown on the diagonal in bold. ^*^ *p* < 0.05, ^**^ *p* < 0.0.

**References**

1. Chen, Y., Tong, Z. & Malkawi, A. Investigating natural ventilation potentials across the globe: Regional and climatic variations. *Build. Environ.* **122**, 386–396 (2017).
2. Ilies, R., Wilson, K. S. & Wagner, D. T. The spillover of daily job satisfaction onto employees’ family lives: The facilitating role of work-family integration. *Acad. Manage. J.* **52**, 87–102 (2009).
3. Schaufeli, W. B., Salanova, M., González-Romá, V. & Bakker, A. B. The measurement of engagement and burnout: A two sample confirmatory factor analytic approach. *J. Happiness Stud.* **3**, 71–92 (2002).
4. Podsakoff, P. M., MacKenzie, S. B., Moorman, R. H. & Fetter, R. Transformational leader behaviors and their effects on followers’ trust in leader, satisfaction, and organizational citizenship behaviors. *Leadersh. Q.* **1**, 107–142 (1990).
5. Baer, M. D. *et al.* Uneasy lies the head that bears the trust: The effects of feeling trusted on emotional exhaustion. *Acad. Manage. J.* **58**, 1637–1657 (2015).
6. Wargocki, P., Wyon, D. P., Baik, Y. K., Clausen, G. & Fanger, P. O. Perceived air quality, sick building syndrome (SBS) symptoms and productivity in an office with two different pollution loads. *Indoor Air* **9**, 165–179 (1999).
7. Lan, L., Wargocki, P., Wyon, D. P. & Lian, Z. Effects of thermal discomfort in an office on perceived air quality, SBS symptoms, physiological responses, and human performance. *Indoor Air* **21**, 376–390 (2011).
8. Underwood, B. J. Paired associate learning: Data on pair difficulty and variables that influence difficulty. *Mem. Cognit.***10**, 610–617 (1982).
9. Porath, C. L. & Erez, A. Does rudeness really matter? The effects of rudeness on task performance and helpfulness. *Acad. Manage. J.* **50**, 1181–1197 (2007).
10. Erez, A. & Isen, A. M. The influence of positive affect on the components of expectancy motivation. *J. Appl. Psychol.* **87**, 1055–1067 (2002).
11. Mannucci, P. V. & Perry-Smith, J. E. “Who are you going to call?” Network activation in creative idea generation and elaboration. *Acad. Manage. J.* **65**, 1192–1217 (2022).
